# Vangl2 interaction plays a role in the proteasomal degradation of Prickle2

**DOI:** 10.1038/s41598-019-39642-z

**Published:** 2019-02-27

**Authors:** Tadahiro Nagaoka, Mikio Furuse, Toshihisa Ohtsuka, Kunihiro Tsuchida, Masashi Kishi

**Affiliations:** 1 Division for Therapies Against Intractable Diseases, Institute for Comprehensive Medical Science, Fujita Health University, Toyoake, Aichi 470-1192 Japan; 2 Division of Cell Structure, National Institute for Physiological Sciences, Okazaki, Aichi 444-8787 Japan; 3 Department of Biochemistry, Faculty of Medicine, University of Yamanashi, Yamanashi, 409-3898 Japan; 4 Neuroscience Laboratory, Research Institute, Nozaki Tokushukai Hospital, Daito, Osaka 574-0074 Japan

## Abstract

The PET and LIM domain-containing protein, Prickle, plays a key role in planar cell polarity (PCP) in *Drosophila*. It has been reported that mutations in the *PRICKLE2* gene, which encodes one of the human orthologues of Prickle, are associated with human diseases such as epilepsy and autism spectrum disorder. To develop preventive and therapeutic strategies for these intractable diseases, we studied the regulation of Prickle2 protein levels in transfected HEK293T cells. Prickle2 levels were negatively regulated by a physical interaction with another PCP protein, Van Gogh-like 2 (Vangl2). The Vangl2-mediated reduction in Prickle2 levels was, at least in part, relieved by proteasome inhibitors or by functional inhibition of the Cullin-1 E3 ubiquitin ligase. Furthermore, the expression of Vangl2 enhanced the polyubiquitination of Prickle2. This ubiquitination was partially blocked by co-expression of a ubiquitin mutant, which cannot be polymerised through their Lys48 residue to induce target proteins toward proteasomal degradation. Together, these results suggest that Prickle2 is polyubiquitinated by the Vangl2 interaction in a Cullin-1-dependent manner to limit its expression levels. This regulation may play a role in the local and temporal fine-tuning of Prickle protein levels during PCP signal-dependent cellular behaviours.

## Introduction

Many epithelial cells display a level of asymmetric distribution of cellular structures within the cell along an axis perpendicular to the apical-basal axis. If the direction of this asymmetry is coordinated among a group of cells within the plane of epithelium, this polar tissue organisation is referred to as planar cell polarity (PCP)^1–4^. PCP is developed by signalling cascades that originate from its core regulatory proteins. If any of these core proteins are missing, the PCP does not develop properly, resulting in a disorganised tissue organisation in the affected epithelia. The core regulators of PCP reside at the cell-cell boundaries, and with the exception of the Flamingo/Celsr family of atypical cadherins, they are localised at either side of the adhesion site. This uneven distribution is thought to give rise to both molecular asymmetry and polarised domains within the cells to allow for subsequent morphological polarisation. In segregating the cellular components to generate asymmetry, the PCP regulators are thought to participate in both positive and negative feedbacks to align their localisation within, and between, neighbouring cells along the prospective PCP axis. The molecular mechanism that generates and controls these feedback loops is not fully understood.

The PCP regulatory proteins are thought to function by forming a protein complex at the plasma membrane, at a sub-membrane region, or at cell-cell junctions. The Frizzled serpentine receptor and the DIX and DEP domain-containing intracellular protein Dishevelled form a protein complex on one side^5^, and the protein complex formed between Van Gogh (Vang)/Strabismus and Prickle locates at the other side^6^. The atypical cadherin Flamingo/Celsr resides on both sides of the cell-cell junctions to function as homophilic cell adhesion molecule required for PCP signalling^7^. Prickle is a PET and LIM domain-containing protein^8^ that is prenylated at a carboxyl-terminal CAAX motif, where cysteine is the site of prenylation^9^, A is a given aliphatic amino acid, and X determines the type of the attached prenyl group. For the Prickle protein, this lipid modification is farnesylation and is thought to be required for its membrane association^10^. Vang tetraspanin physically interacts with Prickle through the carboxyl terminal intracellular region^6,11^, and is thought to recruit the membrane-associated Prickle to the cell-cell junctions. The physiological roles of the farnesylation of Prickle, and of the protein-protein interaction between Prickle and Vang, are however still ambiguous. For instance, Prickle becomes more cytoplasmic in epithelial cells of *Drosophila* Vang mutant^6,10,11^. It has been also demonstrated that expression of a Prickle construct that is unable to be farnesylated rescues the PCP phenotype in the ommatidial rotation of *Drosophila* Prickle mutant^6^.

Although the regulators of PCP are required to be correctly localised by protein modification and/or other transport proteins, this in itself is not sufficient for the development of PCP. In addition, the protein levels need to be tightly regulated; an excess of any one PCP factor may disrupt the negative feedback by interfering with or excluding the competitor proteins more than required, or may result in too much formation and subsequent spread of the functional PCP complex beyond its normal location. For example, to mediate PCP signalling in *Drosophila*, the Dishevelled protein needs to be negatively regulated by Cullin-3/Diablo/Kelch E3 ubiquitin ligase-dependent proteasomal degradation^12^. The E3 complex recognises and ubiquitinates the excess Dishevelled proteins recruited to the junctions by Frizzled. If the levels of Dishevelled protein are increased as a result of defective degradation in mutant tissues, this promotes the accumulation of all the other core proteins, leading to an aberrant development of PCP. Prickle, another intracellular factor localised on the other side of the planar polarised cells, is also degraded by the proteasome; Vang recruits the farnesylated Prickle protein to the asymmetric PCP complex, but at the same time limits the accumulation of excess Prickle through its Cullin-1-dependent ubiquitination^10,13^.

In mammals, genetic mutations of either of the Prickle orthologues are known to give rise to a variety of malformations and disorders. In the early development of neural tissues, PCP signalling regulates the convergence and extension movements of the neural plate, and is required for the normal closure of the neural tubes. In a genetic study in human patients, independent heterozygous missense variants in the *PRICKLE1* gene have been identified in 7 of 810 patients with a variety of neural tube defects^14^. In developed neural circuits, the *Prickle2* gene product is found at the postsynapse^15^, and is required for the normal development of excitatory synapses^16^. In human patients, heterozygous mutations in either the *PRICKLE1* or *PRICKLE2* genes are associated with myoclonic epilepsy^17^. In addition, heterozygous mutations in the *PRICKLE2* gene have been reported in two independent familial cases of autism spectrum disorder (ASD)^18^. In both reports, mice lacking the *Prickle* gene exhibited similar abnormal behaviours to the patients, indicating that the mutations were the causes of the human disorders.

To develop new preventive and therapeutic strategies for these disorders, it is therefore important to understand the molecular mechanisms that control the protein levels of Prickle in mammals. So far, Smurf family ubiquitin ligases have been shown to target Prickle proteins for their ubiquitin-mediated degradation; mice lacking Smurf-1 and -2 display PCP defects in cochlear organisation and in neural tube closure^19^. In contrast, the role of the Cullin-1-mediated degradation of the Prickle protein in the development of PCP, previously demonstrated in *Drosophila* studies^10,13^, has not yet been examined in mammals. In this report, we explored the role of the physical interaction with Vangl2 and that of the Cullin-1 ubiquitin ligase in the negative regulation of Prickle2 levels. We further demonstrated that Prickle2 is polyubiquitinated by the Vangl2 interaction. Our simple transfection assay should be a useful tool for future drug discovery to treat human disorders or congenital anomalies that are related to aberrant expression of Prickle proteins.

## Results

### Vangl2 reduces the protein levels of Prickle2 dependently on the Prickle-binding domain

**Figure 1:**
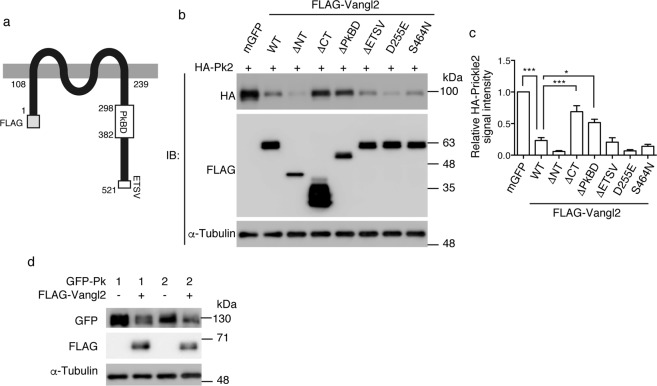
Vangl2 reduction of Prickle levels. (**a**) A schematic presentation of the molecular structure of mouse Vangl2. All Vangl2 constructs used in this study were FLAG-tagged at the amino-terminus. The numerals indicate the number of amino acid residues from the amino-terminal end. Vangl2 has amino-terminal (1–108) and carboxyl-terminal (239–521) intracellular domains. The Prickle-binding domain (PkBD; 298–382) is the main region required for the normal interaction with Prickle2. The ETSV (518–521) sequence at the carboxyl terminal end corresponds to the consensus motif for PDZ domain-interaction. (**b**) Western blot analysis of Prickle2 levels in the presence or absence of the Vangl2 constructs. A HA-tagged Prickle2 expression plasmid was co-transfected with Vangl2 constructs lacking the amino-terminus (ΔNT), carboxyl-terminus (ΔCT), PkBD (ΔPkBD) or PDZ-binding motif (ΔETSV). The effects of co-expression of the membrane-attached GFP (mGFP; control), wild type Vangl2 (WT), as well as loss-of-function mutants of Vangl2 (*Lpt*; D255E, S464N) were also examined. Total lysates of the transfected HEK293T cells were subjected to western blot analysis using anti-HA, anti-FLAG, or anti-α-tubulin antibodies. (**c**) Bar graphs showing the quantitation of the signal intensities of the western blots shown in (**b**). The average Prickle2 levels when co-transfected with a control plasmid (mGFP) was set as one arbitrary unit on the Y-axis. Significant differences versus control groups calculated using one-way ANOVA (p < 0.0001) followed by Tukey’s multiple comparisons test are marked with * (*p < 0.05, **p < 0.01, ***p < 0.001). a.u.: arbitrary unit. (**d**) Western blot analysis of the protein levels of Prickle1 and 2. Total lysates of cells transfected with the indicated expression plasmids were subjected to western blot analysis using anti-HA, anti-FLAG, or anti-α-tubulin antibodies. pCAG-mGFP (Addgene #14757) was mixed as the control vector to adjust the total amount of the transfected plasmid DNA (**d**). Note the similar decrease in Prickle1 levels induced by Vangl2.

In our previous study, the protein levels of Prickle2 were found to be highly decreased following the co-transfection of Vangl2 in the presence of PSD-95^20^. Prickle2 is capable of forming a protein complex with PSD-95 through Vangl2; therefore, using a western blot analysis of the total cell lysates, we first addressed whether this reduction of Prickle2 levels requires PSD-95. In transfected HEK293T cells, which essentially do not express endogenous Prickle1 or Prickle2 (Supplementary Fig. S1), Prickle2 levels were reduced by Vangl2, even in the absence of PSD-95 (Fig. 1b; 2nd lane, 22.8 ± 6.5%, P < 0.0001). Furthermore, using deletion constructs of Vangl2, we determined which domains of Vangl2 are required for the reduction in Prickle2 levels^16,21^ (Fig. 1a–c). Vangl2 is a tetraspanin with intracellular amino- and carboxyl-terminal domains, and its Prickle-binding domain (PkBD) is within the carboxyl-terminus, all of which are more than 99% conserved in mice and humans. Although a Vangl2 construct lacking the amino-terminal region still decreased Prickle2 levels (Fig. 1b; 3rd lane, 5.7 ± 1.9%, P < 0.0001), those without the Prickle-binding domains (Fig. 1b; 4th and 5th lanes) did not reduce the Prickle2 levels to the same extent as wild-type Vangl2 (ΔCT; 74.7 ± 10.4%, P = 0.155 vs. mGFP^22^ and P = 0.0005 vs. WT Vangl2. ΔPkBD; 53.8 ± 7.0%, P = 0.0018 vs. mGFP and P = 0.049 vs. WT Vangl2). Furthermore, point mutations of the conserved amino acid residues located outside of the Prickle-binding domain, that cause loss of membrane targeting, retention at the endoplasmic reticulum, and shortening of half-life of the Vangl2 protein *in vivo* (Fig. 1b; 7th and 8th lanes for D255E^23,24^ and S464N^25,26^, respectively), did not affect Vangl2’s ability to reduce Prickle2 levels (D255E; 7.3 ± 2.4%. S464N; 14.4 ± 4.3%). Finally, deletion of the carboxyl-terminal PDZ-binding motif (ETSV)^27^, which is required for interaction with PSD-95^16,28^, did not restore Prickle2 levels (Fig. 1b; 6th lane, 22.5 ± 9.4%), further confirming that PSD-95 is not involved in this reduction in Prickle2 levels as expected from the data shown in Fig. 1b by Nagaoka *et al*.^20^. These results demonstrate that the protein interaction between Prickle2 and Vangl2 is critical for reduction in Prickle2 levels.

In mammals, another Prickle family protein, Prickle1^29^, is known to be homologous with Prickle2 having the same domain structure and molecular weight. We therefore examined whether the protein levels of Prickle1 are also affected by Vangl2 using the same experimental approach. Prickle1 levels were similarly reduced by co-transfection with Vangl2 (Fig. 1d). This result suggests that the reduction in Prickle protein levels by Vangl is conserved among the mammalian paralogues.

### Vangl2 decreases the protein levels of Prickle2 through the proteasome

**Figure 2:**
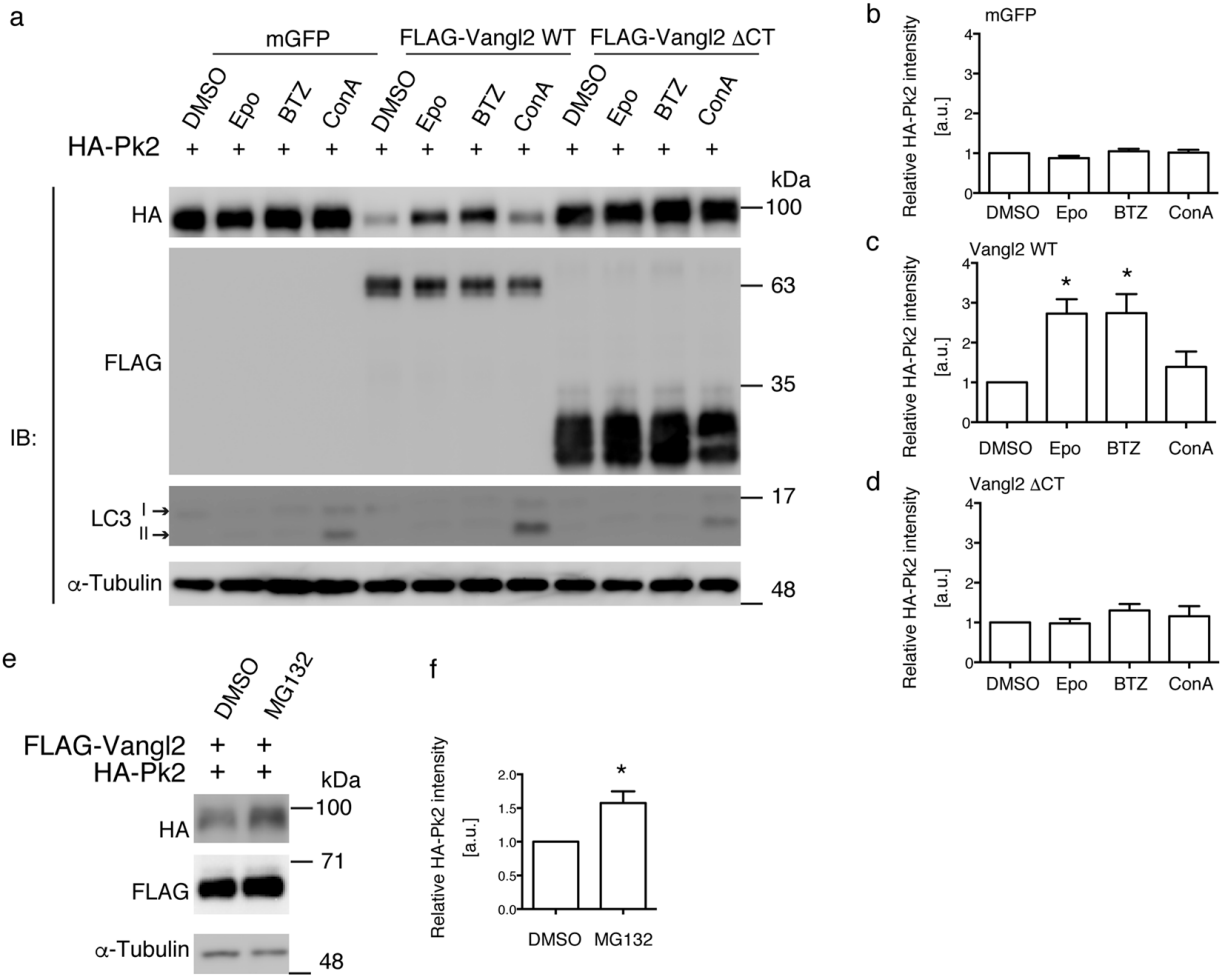
Effects of proteasome or lysosome inhibitors on the Vangl2-induced reduction of Prickle2 levels. (**a**,**e**) Western blot analysis of Prickle2 levels in lysates of HEK293T cells transfected with the indicated expression plasmids or in cells treated with the indicated inhibitors. Note the partial recovery of Prickle2 levels following treatment with epoxomicin (Epo) and bortezomib (BTZ) (**a**) or MG132 (**e**) but not by concanamycin A (ConA) (**a**) in cells expressing the wild type Vangl2. (**b**–**d**,**f**) Bar graphs showing the quantitation of the signal intensities of the western blots shown in (**a**,**e**). The plasmids co-transfected with HA-Prickle2 were mGFP (b: control), WT Vangl2 (**c,f**), or Vangl2ΔCT (**d**). The average Prickle2 levels in cells treated with vehicle (DMSO) was set as one arbitrary unit on each Y-axis. Note the specific effects of the proteasome inhibitors on cells expressing the wild type Vangl2 (**c**,**f**). Significant differences (p < 0.05) versus control groups calculated using one-way ANOVA followed by Tukey’s multiple comparisons test (**b**–**d**) or by Student’s t test (**f**) are marked with *a.u.: arbitrary unit.

Because it is known that Vang-dependent proteasomal degradation of the Prickle protein is required for the normal development of PCP in *Drosophila*, we examined whether the reduction in Prickle2 levels by Vangl2 in our assay system was also a proteasomal event. Thirty-two hours after transfection with the indicated expression plasmids, cells were treated for 16 h with a variety of different agents that suppress protein degradation (dissolved in DMSO and added to the culture medium), after which the cell lysates were harvested for western blot analysis. Treatment with the proteasome inhibitors epoxomicin or bortezomib^30^, clearly restored the Prickle2 levels reduced by Vangl2 (6th and 7th lanes of Fig. 2a,c; 2.7 ± 0.4 and 2.7 ± 0.5, respectively, one-way ANOVA P = 0.0074). In contrast, treatment with concanamycin A (ConA), an inhibitor of vacuolar-type H^+^-ATPase (V-ATPase) activity, which results in the suppression of the lysosomal protein degradation^31^, did not restore the reduced Prickle2 levels (8th lane of Fig. 2a,c; 1.4 ± 0.4), indicating that the Vangl2-mediated reduction in Prickle2 levels is not mediated through lysosomal degradation. Furthermore, Prickle2 levels were relatively unaffected by epoxomicin or bortezomib treatment in the absence of co-transfected wild type Vangl2 (1st-4th and 9th-12th lanes of Fig. 2a,b,d; one-way ANOVA P = 0.199 and P = 0.496, respectively), suggesting that the proteasomal regulation of Prickle2 levels is a specific effect related to Vangl2 and not a non-specific mechanism for protein turnover. We also confirmed a similar recovery of Prickle2 levels following treatment with a reversible inhibitor of the proteasome, MG132 (2nd lane of Fig. 2e,f; 1.7 ± 0.2, P = 0.029). The effect of the proteasome inhibitor was time-dependent: the recovery of Prickle2 levels was not observed after 6 h of the BTZ treatment but almost plateaued by 16 h (Supplementary Fig. S2).

### Vangl2 decreases the protein level of Prickle2 through Cullin-1

**Figure 3:**
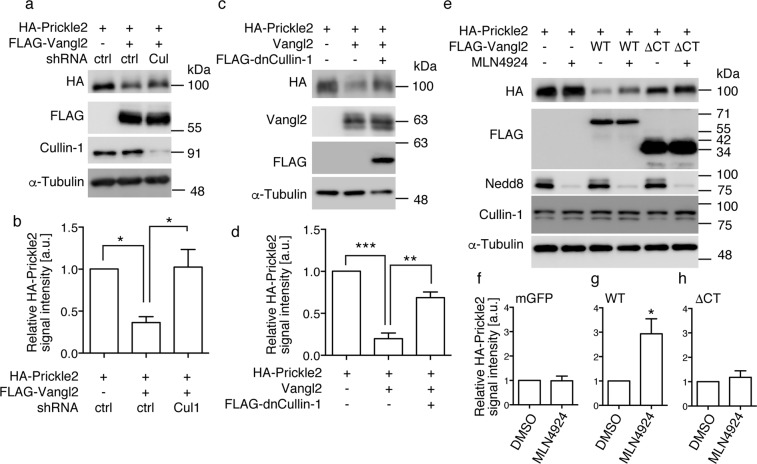
Loss-of-function studies of Cullin-1 in the Vangl2-induced reduction of Prickle2 levels. (**a**,**c**,**e**) Western blot analysis of Prickle2 levels in HEK293T cells co-transfected with shRNA-Cullin-1 (**a**), or an expression plasmid encoding a dominant-negative form of Cullin-1 (**c**). The effect of the Nedd8 inhibitor MLN4924 was also examined (**e**). (**b**,**d**,**f–h**). Bar graphs showing the quantitation of the signal intensities of the western blots shown in (**a**,**c**,**e**). Cells were co-transfected with shRNA-Cullin-1 (**b**), or plasmids encoding dnCullin-1 (**d**), control mGFP (**f**), WT Vangl2 (**g**), and Vangl2ΔCT (**h**). The average Prickle2 expression levels in cells when co-transfected with the control plasmid (**b,d**) or when treated with the vehicle (DMSO) (**f**–**h**) was set as one arbitrary unit on each Y-axis. Note the specific effects of MLN4924 on cells expressing wild type Vangl2 (**g**). pCAG-mGFP (**a,c,e**) and/or pCS2FLAG (Addgene #16331) (**c**) were mixed as the control vector(s) to adjust the total amount of the transfected plasmid DNA. Significant differences versus control groups calculated using Tukey’s multiple comparisons test (**b**,**d**) or Student’s t test (**f**–**h**) are marked with * (*p < 0.05, **p < 0.01, ***p < 0.001). a.u.: arbitrary unit.

In *Drosophila*, it has been shown that the Vang-dependent reduction of Prickle levels requires Cullin-1, which is known to be a scaffold component of the Cullin-Ring type E3 ubiquitin ligase^10^. To address if Cullin-1 is also required for Vangl2-induced reduction of Prickle2 levels, we co-transfected cells with an shRNA-encoding plasmid that effectively suppresses the expression levels of endogenous Cullin-1 protein (Fig. 3a; 3rd lane). Following transfection with the shRNA, the reduced Prickle2 levels caused by Vangl2 expression were significantly restored (3rd lane of Fig. 3a,b; 102 ± 21%, one-way ANOVA P = 0.0174). Similar results were obtained by co-transfection of a dominant-negative form of Cullin-1 (dnCullin-1)^32^ (3rd lane of Fig. 3c,d; 68.7 ± 6.8%, one-way ANOVA P = 0.0002), which lacks its carboxyl-terminal region. Furthermore, treatment with MLN4924, an inhibitor of the Nedd8 modification required for the activation of Cullin-1^33^, restored the Prickle2 levels reduced by Vangl2 (4th lane of Fig. 3e,g; 2.9 ± 0.6, P = 0.035). These results indicate that Cullin-1 is an essential component of the proteasomal pathway through which Vangl2 induces the degradation of Prickle2.

We further examined whether overexpression of Cullin-1 alone affects the Prickle2 levels. Transfection of the expression vector of Cullin-1 did not decrease the expression levels of Prickle2 in contrast to the effect of the Vangl2 overexpression (3rd lane of Supplementary Fig. S3a. Supplementary Fig. S3b; 1.12 ± 0.07). We, however, observed that inhibition of Cullin-1 expression using shRNA in the HEK293T cells increased Prickle2 expression levels (2nd lane of Supplementary Fig. S3c. Supplementary Fig. S3d; 4.3 ± 1.1). These results suggest that Cullin-1, which functions as a scaffold protein, is required, but not rate-limiting, for restricting Prickle2 expression levels.

Cullin-1 is generally considered a component of the SCF complex (Skp, Cullin, F-box containing complex), a multi-protein E3 ubiquitin ligase that catalyses the ubiquitination of proteins destined for proteasomal degradation^34^. To address whether Vangl2-induced degradation of the Prickle2 protein is mediated through the SCF complex, we examined the requirement for Skp1, another component of the SCF complex^34^. Although two independent shRNA constructs that effectively silenced the protein level of endogenous Skp1 were tested, we did not observe the recovery of Prickle2 levels (Supplementary Fig. S4a; 4th and 5th lanes. Supplementary Fig. S4b). In the same experiment, co-transfection with shRNA-Cullin-1 did result in the expected recovery of Prickle2 levels (Supplementary Fig. S4a; 3rd lane. Supplementary Fig. S4b). These data indicate that Skp1 is not critical for the Vangl2-induced reduction in Prickle2 levels in mammalian cells, and that a Cullin-1-containing molecular complex other than SCF may participate in this process^35^.

### Farnesylation of Prickle2 is not essential for its Vangl2-induced degradation in transfected cells

**Figure 4:**
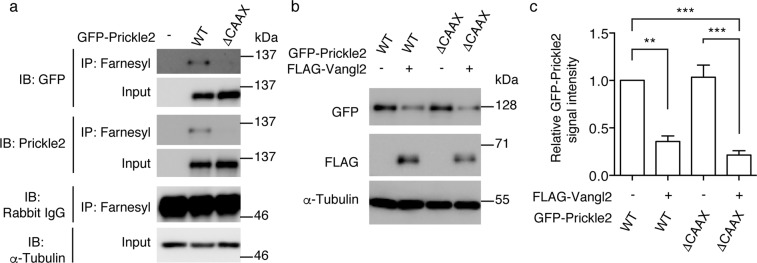
Farnesylation is not required for the Vangl2-induced reduction in Prickle2 levels in transfected HEK293T cells. (**a**) Western blot analysis of the Prickle2 protein immunoprecipitated using anti-farnesyl antibodies. Prickle2 immunoprecipitates with (WT) or without (ΔCAAX) the carboxyl-terminal CAAX motif were analysed via western blot, using anti-GFP or anti-Prickle2 antibodies. Note the lack of immunoprecipitation of the ΔCAAX mutant. (**b**) Western blot analysis of the Prickle2 levels in the presence (WT) or absence (ΔCAAX) of the carboxyl-terminal CAAX motif required for protein farnesylation. (**c**) Bar graphs showing the quantitation of the signal intensities of the western blots shown in (**b**). The average Prickle2 level expressed in cells transfected with wild type Prickle2 were set as one arbitrary unit on the Y-axis. Note the similar reduction of Prickle2 levels by Vangl2 in the presence or absence of the CAAX motif. pcDNA3.1 was mixed as the control vector to adjust the total amount of the transfected plasmid DNA (**a,b**). Significant differences versus the control group calculated using one-way ANOVA (p < 0.0001) followed by Tukey’s multiple comparisons test are marked with * (**p < 0.01, ***p < 0.001). a.u.: arbitrary unit.

The Prickle family of proteins has a carboxyl-terminal CAAX motif, which is the consensus sequence for the post-translational modification, farnesylation^10^. The covalently attached farnesyl group retains the Prickle proteins at the plasma membrane through its hydrophobicity. Deletion of the CAAX motif therefore results in a loss of farnesylation (Fig. 4a), leading to the inability of Prickle proteins to associate with the plasma membrane. In *Drosophila*, membrane attachment through this motif is required for the Prickle protein to be degraded by the Vang-dependent proteasomal event^10^. To address whether this requirement is conserved in mammals, we examined whether a Prickle2 protein lacking the CAAX motif could be degraded following co-transfection with Vangl2. Neither the normal expression levels of Prickle2 (1st and 3rd lanes of Fig. 4b,c; 103 ± 13%) nor the sensitivity of Prickle2 to Vangl2 expression was affected by the deletion of this motif (2nd and 4th lanes of Fig. 4b,c; 35.7 ± 5.9% and 21.5 ± 4.4%, respectively), which eliminated the farnesylation of Prickle2 (Fig. 4a). This result indicates that the Vangl2-induced degradation of Prickle2 does not necessarily require the membrane localisation of Prickle2, at least in transfected HEK293T cells.

### Vangl2 increases Lysine-48-linked polyubiquitination levels on Prickle2

**Figure 5:**
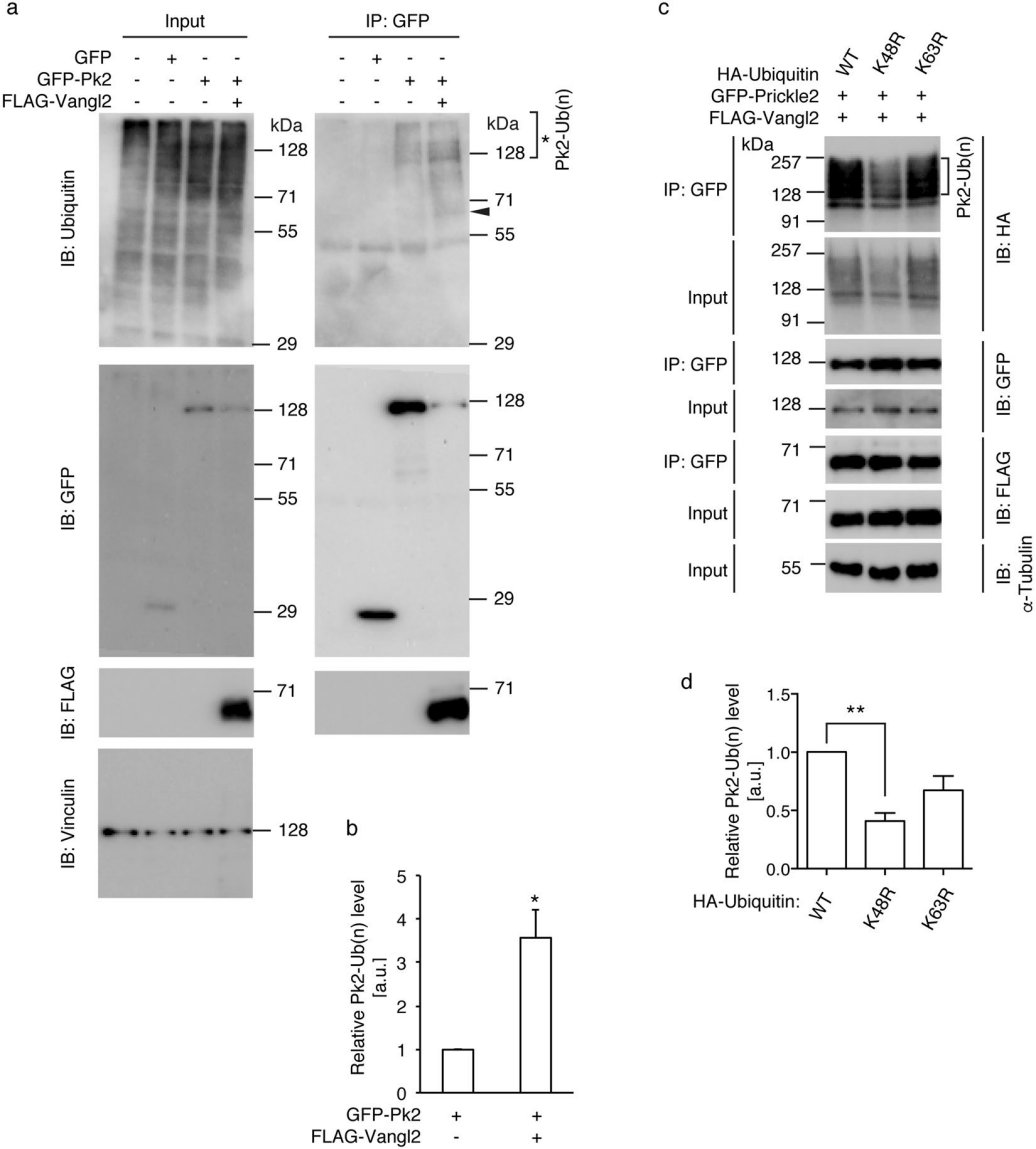
Vangl2 enhances ubiquitination of Prickle2. (**a**,**c**) Cells were transfected with the indicated expression plasmids, and the cell lysates, as well as immunoprecipitates, were subjected to western blot analysis. (**b**,**d**) Bar graphs showing the ratio of the amount of precipitated ubiquitin (parenthesis in a and c, respectively) to that of Prickle2. (**a**) Ubiquitin molecules attached to the immunoprecipitated Prickle2 protein were detected using anti-ubiquitin antibodies. Note that the ratio of ubiquitinated Prickle2 was significantly increased in the presence of Vangl2 (**b**). (**c**) Effects of the introduction of the lysine-mutated ubiquitin monomers. GFP-tagged Prickle2 was immunoprecipitated from HEK293T cells co-expressing wild type (WT), K48R, or K63R mutant forms of HA-tagged ubiquitin monomers. The levels of the different ubiquitins attached to the immunoprecipitated Prickle2 were detected using anti-HA antibodies. pcDNA3.1 was mixed as the control vector to adjust the total amount of the transfected plasmid DNA (**a**). Significant differences versus the control group calculated using Student’s t test (**b**) or one-way ANOVA followed by Tukey’s multiple comparisons test (**d**) are marked with * (*p < 0.05, **p < 0.01). a.u.: arbitrary unit.

Since the Prickle2 levels are markedly reduced by co-transfection with Vangl2 through proteasomal degradation, which is dependent on Cullin-1, it is likely that expression of Vangl2 enhances the ubiquitination of Prickle2. We examined this possibility using western blot analysis of the immunoprecipitated Prickle2 protein. GFP-tagged Prickle2 was co-transfected with or without Vangl2 (Fig. 5a; 4th and 3rd lanes, respectively). The resultant immunoprecipitates using anti-GFP antibodies were analysed. Following extended exposure, several protein bands with a molecular weight of >128 kDa were detected using anti-ubiquitin antibodies, even in the immunoprecipitates from the cell lysates without Vangl2 transfection (Fig. 5a; 3rd lane). At a similar molecular weight, we also found increased levels of anti-ubiquitin signals in the immunoprecipitates from cell lysates with Vangl2 co-transfection (Fig. 5a; 4th lane, asterisk. Fig. 5b). In this experiment, Prickle2 expression levels in the total cell lysates were markedly reduced (Fig. 5a; 4th lane), indicating that Vangl2 enhanced the ubiquitination and degradation of Prickle2. Ubiquitination of the GFP tag itself was neither detected nor enhanced by Vangl2 co-transfection (Supplementary Fig. S5). We further confirmed that ubiquitination of FLAG-tagged Prickle2 is also enhanced by Vangl2 (3rd lane of Supplementary Fig. S6; 2.2 ± 0.3). These results support the notion that Vangl2 enhances the ubiquitination and subsequent degradation of Prickle2. In the GFP-Prickle2 immunoprecipitates, we noticed that a 68 kDa protein band, corresponding to Vangl2 protein, was detected using anti-ubiquitin antibodies (Fig. 5a; 4th lane. arrowhead). Thus, we reasoned that Vangl2 is, to some extent, ubiquitinated in the Vangl2/Prickle2 complex.

In protein ubiquitination, ubiquitin monomers are tandemly added to the target proteins through the repetitive amide bonds between their specific lysine or methionine residues and carboxyl terminal glycine residues. The polyubiquitin chains formed through lysine-48 in the ubiquitin monomers generally target proteins for proteasomal degradation^36^. In contrast, if the ubiquitin chains are polymerised through their lysine-63 residues, the polyubiquitin chains usually function as molecular moieties that can modulate protein-protein interactions^37^. Because lysine-48 and lysine-63 residues are indispensable for each type of polymerisation, the introduction of ubiquitin monomers that have arginine substitution at these lysine residues (K48R and K63R, respectively) inhibit the formation of the respective polyubiquitin chains. GFP-tagged Prickle2 was co-transfected with Vangl2 and a plasmid containing either of these two ubiquitin constructs, after which Prickle2 was immunoprecipitated and the immunoprecipitates analysed by western blotting. As expected, Prickle2 co-transfected with Vangl2 and wild type ubiquitin was modified with HA-tagged ubiquitin^38^ (Fig. 5c; 1st lane). However, if ubiquitin construct encoding the K48R substitution was transfected instead, the levels of ubiquitin covalently attached to Prickle2 were significantly reduced (2nd lane of Fig. 5c; 41.0 ± 6.8%, one-way ANOVA P = 0.0052. Fig. 5d). In contrast, when the construct encoding the ubiquitin monomer with the K63R substitution was used instead, the overall ubiquitin levels on Prickle2 were not significantly altered (3rd lane of Fig. 5c; 67.3 ± 12.3%, one-way ANOVA P = 0.135. Fig. 5d). These results indicate that the Vangl2-induced ubiquitination of Prickle2 is mediated through lysine-48-dependent polyubiquitination, which in most cases targets proteins for proteasomal degradation. This result is consistent with the notion that the Vangl2 enhancement of polyubiquitination on Prickle2 is important in controlling Prickle2 levels.

### Vangl2 is not a sole regulator of the expression levels of Prickle2 protein in mice

Our results suggest that Vangl2 interaction plays a role in the proteasomal degradation of Prickle2. It is, however, still unknown whether Vangl2 is essential for this process: there might be other factors that are redundant for this function of Vangl2. To confirm whether Vangl2 is required for the negative regulation of Prickle2, we analyzed Prickle2 expression levels in brain tissue lysates derived from *Looptail* mice harboring a loss-of-function mutation in the Vangl2 gene. Western blot analysis showed that these mice exhibited markedly reduced expression levels of the Vangl2 protein (2nd lane of Supplementary Fig. S7a; WT:1.0 ± 0.0, *Lp*:0.40 ± 0.16. Supplementary Fig. S7b) due to the shortened half-life^25^ and its dominant-negative action^39^. Prickle2 levels were, however, not significantly changed in the tissue lysates: we did not observe increased expression of Prickle2 (2nd lane of Supplementary Fig. S7a; WT:1.0 ± 0.2, *Lp*:1.1 ± 0.2. Supplementary Fig. S7c). This result indicated that there are molecules other than Vangl2 which redundantly function to restrict Prickle2 levels in the mouse brain. Therefore, we reasoned that there are more complex mechanisms in mammals than in *Drosophila* for regulating the Prickle protein.

## Discussion

In order to develop cell polarity, it is generally accepted that the expression levels of regulatory proteins need to be tightly regulated, both locally and temporally. Prickle2 is a mammalian homologue of the *Drosophila* Prickle protein that is required for the normal development of PCP. Previous studies on *Drosophila* have shown that Prickle protein levels in PCP are negatively regulated by Cullin-1- and Vang-dependent ubiquitination^10^. Although our data show that Vangl2 does, in fact, induce the ubiquitination and subsequent degradation of the Prickle2 protein in a Cullin-1-dependent manner, we could not confirm the requirements for the farnesylation of Prickle2^10^ and the need for the Skp1 ubiquitin ligase^13^, both of which have been genetically demonstrated in *Drosophila* studies. Our data neither indicated that endogenous Vangl2 is required for the downregulation of Prickle2 in the mouse brain. Vangl proteins may not be necessarily involved or other molecules may play similar roles *in vivo*. Further studies are required to settle the discrepancies found between our data and those reported in *Drosophila* studies. The requirement of Vangl1 and Skp1 for the regulation of Prickle2 levels will be genetically examined using mutant mouse strains in the near future.

Prickle is also known to be transported and aggregated by Vang^6,10,11^. The molecular mechanism that switches Vang’s action on Prickle from transport to ubiquitination remains to be elucidated. If the degradation is required for the removal of excess molecules, the local concentration of the Prickle protein itself might be the inducer of this transition. In this regard, it is noteworthy that Prickle proteins self-multimerises through the same protein region required for the interaction with Vang^6^. So far, it has been not determined whether these protein-binding events are cooperative or exclusive, and also, the affinities of the Vang-Prickle and Prickle-Prickle protein interactions have not been measured or compared. However, if the Vang-Prickle complex is in a high concentration locally, Prickle proteins stochastically detached from the Vang-Prickle complexes should also be in higher concentration. This may make it easier for them to self-multimerise to block the reformation of the Vang-Prickle complex. Prickle proteins that remain unbound to Vang may become vulnerable to proteasomal degradation. Future studies using purified proteins, as well as molecular imaging should reveal the exact mechanism that degrades excess Prickle2 proteins dependent on its physical interaction with Vangl2.

In the mammalian nervous systems, the Prickle2 protein, as well as the Vangl2 protein, is known to be localised at the postsynaptic density (PSD), which is an electron-dense cellular structure of neurons containing critical molecules for the neurotransmissions^15,16,20,28,40,41^. One of the most notable protein found in the PSD is PSD-95^42^, a PDZ, SH3, and MAGUK domain-containing molecular scaffold protein that accumulates synaptic molecules such as NMDA-type glutamate receptors^43,44^ and Neuroligins^45,46^ through the PDZ interaction. Vangl2 is another PDZ interactor of PSD-95 that can be immunoprecipitated with the PSD^16^, or other synapse-rich sub-fraction of mouse brain lysates^28^. Prickle2 can form a protein complex with PSD-95 through Vangl2^20^, indicating the possibility that Prickle2 directly affects not only synaptic development, but also transmission and plasticity. Several heterozygous mutations in either the Prickle1 or Prickle2 genes have been reported in patients with epilepsy^17^. Each Prickle2 protein with a missense mutation found in patients has been shown to be functionally abnormal in the convergence-extension movement and the calcium release frequency in zebrafish embryos, which are related to the Wnt/PCP and Wnt/calcium signalling, respectively. Although the molecular mechanisms by which Prickle gene mutations cause epilepsy have not been elucidated, the mutant phenotype is common to mouse and *Drosophila*. In addition, two independent mutations in the Prickle2 gene have been found in familial mental illness in which both offspring were diagnosed as having ASD^18^, which is frequently accompanied with epilepsy. Interestingly, mice lacking the Prickle2 gene show an abnormal behaviour similar to human autism. Furthermore, while a wild-type construct encoding human Prickle2 was able to complement the morphological and electrophysiological phenotypes observed in Prickle2-deficient mouse neurons, two Prickle2 constructs harbouring the same point mutations found in patients could not. Since these phenotypes are due to a reduced expression of the wild-type Prickle2 protein, the degradation of Prickle2 might be a reasonable target for development of therapeutic and preventive strategies to treat human epilepsy and ASD. Future efforts to identify drugs that specifically inhibit the degradation of Prickle2 could help overcome those incurable diseases^47^.

## Methods

### Chemicals

All chemicals were purchased from Wako Pure Chemical Industries, Ltd. (Osaka, Japan), Nacalai Tesque, Inc. (Kyoto, Japan) or Sigma-Aldrich Co. LLC (St. Louis, MO, USA) unless otherwise stated.

### Plasmids and antibodies

The PDF file of the Supplemental Information contains detailed information on the plasmids and antibodies used in this study (Supplementary Tables S1 and S2, respectively). All the newly constructed plasmids were sequenced to confirm the absence of sequence errors due to the PCR amplification or oligonucleotide synthesis.

### Tissue culture

HEK293T cells (American Type Culture Collection, Manassas, VA, USA) were cultured under standard conditions in Dulbecco’s modified Eagle’s medium containing GlutaMAX (Life Technologies, Grand Island, NY, USA) supplemented with 10% foetal bovine serum (BioWest LLC, Kansas City, MO, USA) and penicillin-streptomycin (Life Technologies) at 37 °C in the presence of 5% CO_2_ in a water-jacketed incubator. Plasmid DNA transfections were performed with Lipofectamine 2000 (Life Technologies) according to the manufacturer’s instructions. The total amount of DNA used for each transfection was adjusted to 3.0 μg (three different kinds of plasmid) or 2.0 μg (two different kinds of plasmid) for each well of a 6-well plate using control vectors indicated in the figure legends. Unless otherwise stated, the cells were harvested for biochemical analysis after 24 h of transfection, and the different inhibitors were applied 16 h before the cell harvest. For the time course study of the proteasome inhibitor, BTZ was applied for the indicated time periods just before cell harvesting. For experiments using shRNA, FCN or FSΔV vector with scrambled shRNA sequence was used as the control^16,20^, and the cells were harvested after 72 h of transfection. The inhibitors were used at the following concentrations; MG132 (10 μM), bortezomib (10 μM), epoxomicin (5 μM; Peptide Institute, Osaka, Japan), concanamycin A (50 nM), MLN4924 (2 μM; R&D Systems, Minneapolis, MN, USA).

### Immunoprecipitation and WB analysis

Immunoprecipitation and WB analyses were performed as described^16^. HEK293T cells were seeded on 6-well plates at 5 × 10^5^ cells/well. The cells were harvested in 350 μl ice-cold cell lysis buffer (1% NP-40, 150 mM NaCl, 50 mM Tris–Cl pH 7.5, and 10% glycerol, supplemented with protease inhibitors from Roche). The cell lysate (300 μl) was immunoprecipitated overnight at 4 °C with the indicated amount of antibodies and 10 μl of Protein G Mag Sepharose Xtra (GE Healthcare Life Sciences, Pittsburgh, PA, USA) slurry. The Sepharose beads were washed 5 times with the lysis buffer and boiled in 2 × Laemmli sample buffer (Bio-Rad) supplemented with 0.1 M DTT. Approximately quarter of the supernatant was subjected to SDS–PAGE, and a total of 1% of the cell lysate used for each experiment was loaded as the input. Protein samples separated using SDS–PAGE were transferred to Immobilon-P PVDF membranes (Millipore), blocked with Tris-buffered saline pH 7.6 (TBST; 20 mM Tris, 137 mM NaCl, 0.1% Tween-20, and 5% skim milk) for 2 h, incubated with the primary antibodies diluted in TBST overnight at 4 °C, and then incubated with the secondary antibodies conjugated to horseradish peroxidase for 2 h. After 5 times of the final TBST wash without skim milk, the membranes were subjected to chemiluminescent reaction, using Western Blotting Substrate Plus (Thermo Fisher Scientific, Waltham, MA, USA) or ImmunoStar LD (Wako Pure Chemical Industries). As for the western blot with anti-ubiquitin antibodies, the PVDF membranes are denatured using 6 M Guanidine hydrochloride, 1 mM Phenylmethylsulfonyl fluoride, 10 mM N-ethylmaleimide, 5 mM mercaptoethanol, 20 mM Tris-HCl pH 7.5 for 30 min at 4 °C. The signal intensities were calculated using ImageJ.

### Animals

*Loop-tail* mutants of the *LPT/Le* stock were obtained from the Jackson Laboratory (#000220) and backcrossed to C57BL/6 J mice for at least six generations. The Animal Use and Care Committees of Fujita Health University and Nozaki Tokushukai Hospital authorised all animal experiments, which were performed in accordance with the National Institutes of Health Guidelines for Care and Use of Laboratory Animals.

### Statistical analysis

All of the measurements were conducted in a blinded fashion. Each experiment was repeated at least three times and essentially produced consistent results. The measured values from three independent experiments were used for each statistical analysis using Prism 6 (GraphPad Software, La Jolla, CA, USA) or Excel 2016 (Microsoft Corporation, Redmond, WA, USA). The average ratio of one of the samples was set as one arbitrary unit in order to represent the relative amounts, and this was used as the standard in the statistical analysis. In the graphs, error bars are shown to indicate standard error of the mean.

## Supplementary information

Supplementary Information

## Data Availability

The datasets during and/or analysed during the current study available from the corresponding author on reasonable request.
