# Supplementary material for: Vangl2 interaction plays a role in the proteasomal degradation of Prickle2
Source: Sci Rep. 2019 Feb 27;9:2912. doi: 10.1038/s41598-019-39642-z (PMC6393536; doi:10.1038/s41598-019-39642-z)
Supplement: Supplementary file 1 — Supplementary Information [file 41598_2019_39642_MOESM1_ESM.pdf]

Supplementary Information

Vangl2 interaction plays a role in the proteasomal degradation of Prickle2

Tadahiro Nagaoka[1], Mikio Furuse[2], Toshihisa Ohtsuka[3], Kunihiro Tsuchida[1] & Masashi Kishi[4]

[1]Division for Therapies Against Intractable Diseases, Institute for Comprehensive Medical Science, Fujita Health University, Toyoake, Aichi 470-1192, Japan
[2]Division of Cell Structure, National Institute for Physiological Sciences, Okazaki, Aichi 444-8787, Japan
[3]Department of Biochemistry, Faculty of Medicine, University of Yamanashi, Yamanashi 409-3898, Japan
[4]Neuroscience Laboratory, Research Institute, Nozaki Tokushukai Hospital, Daito, Osaka 574-0074, Japan

Supplementary Figures

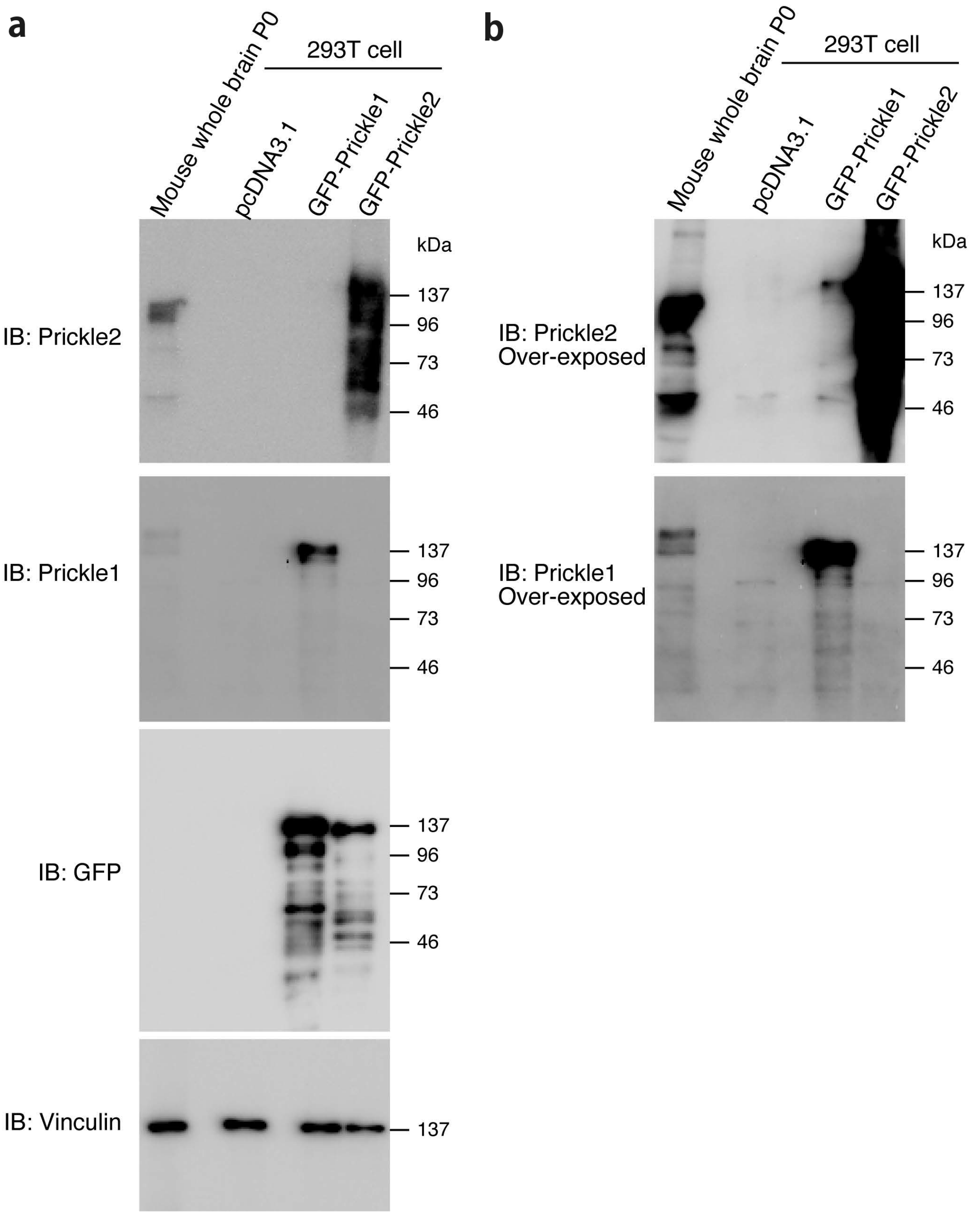

Supplementary Figure S1

Western blot analysis of Prickle1 and Prickle2. Whole cell lysates of HEK293T cells transfected with GFP-Prickle1 (3rd lanes) or GFP-Prickle2 (4th lanes) plasmid were subjected to western blot analysis using anti-Prickle2 or anti-Prickle1 antibodies. (a) Each blot was exposed until the respective protein band in the whole brain lysates derived from postnatal day 0 mice became visible (1st lanes). Even on the overexposed blots (b), endogenous Prickle1 or Prickle2 protein was undetectable in non-transfected HEK293T cell lysates (2nd lanes). pcDNA3.1 was mixed as the control vector to adjust the total amount of the transfected plasmid DNA.

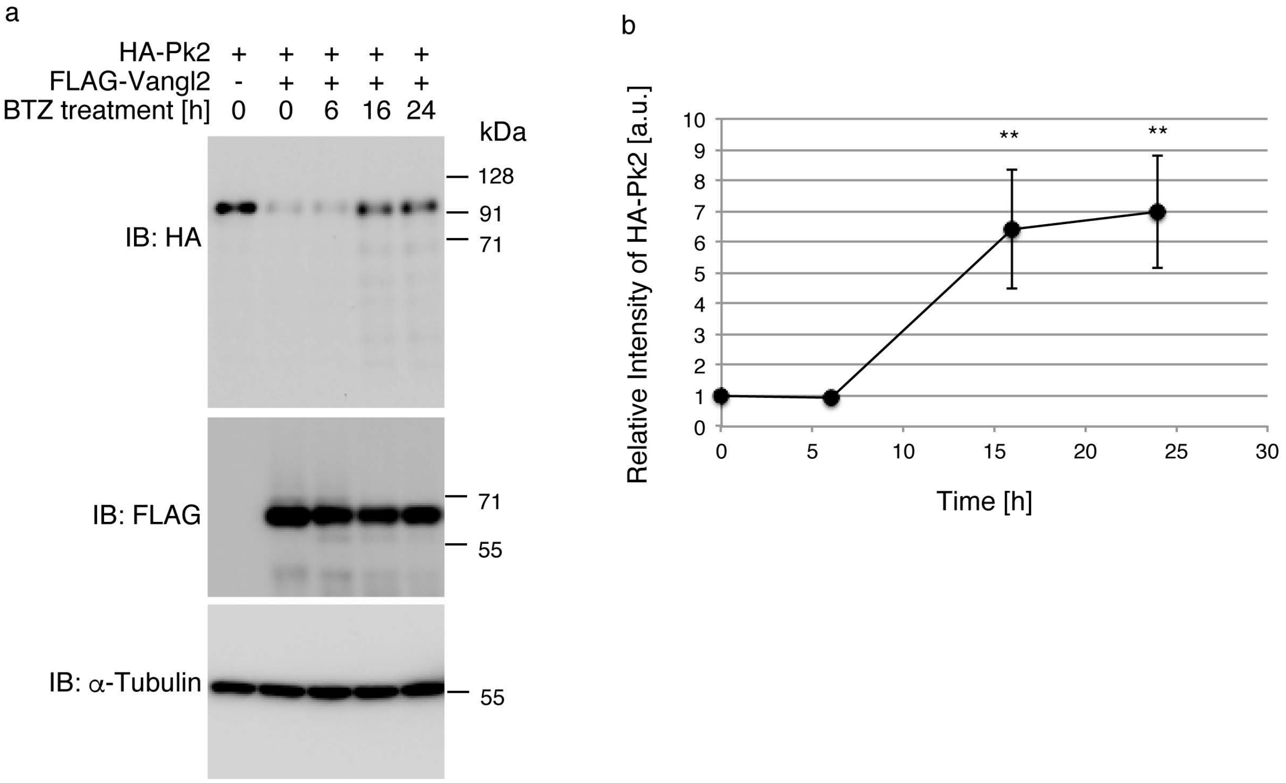

Supplementary Figure S2

Time-dependent recovery of Prickle2 levels by the proteasome inhibitor. (a) Prickle2 expression levels were determined by western blot analysis using anti-HA tag antibodies. The HEK293T cells were treated with BTZ for the indicated time period just before cell harvest. (b) A line graph showing the time course of Prickle2 levels recovered by BTZ treatment (6 h; 0.93±0.13, 16 h; 6.4±1.9, 24 h; 7.0±1.8). The intensity of the Prickle2 chemiluminescent signals was determined using ImageJ. The average Prickle2 levels without BTZ treatment were set as one arbitrary unit on the Y-axis. pCS2FLAG was mixed as the control vector to adjust the total amount of the transfected plasmid DNA (a). Significant differences versus the control group were calculated using Student's t test are marked with ** (**; $p<0.01$). a.u.: arbitrary unit.

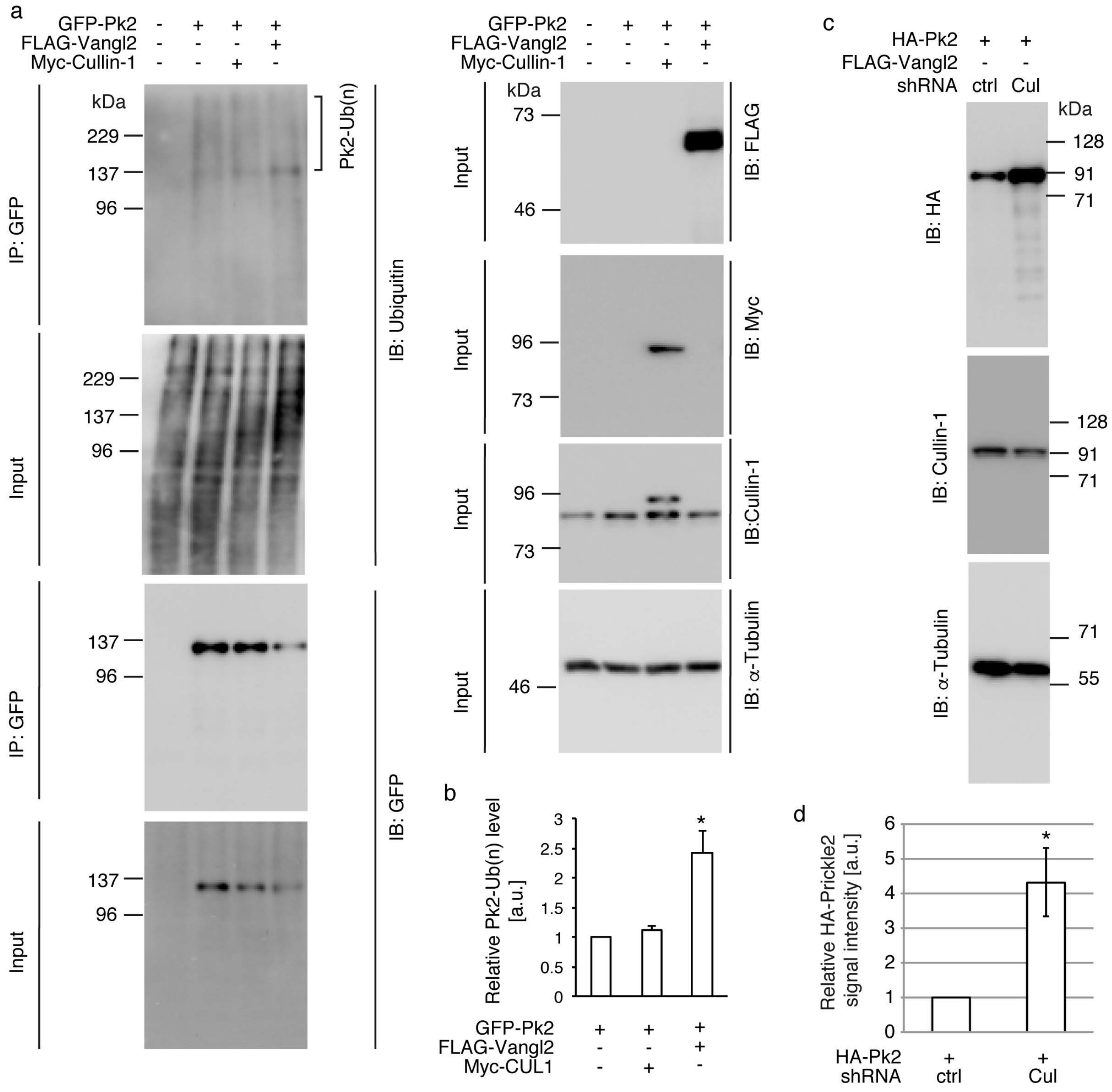

Supplementary Figure S3

Cullin-1 is not the rate-limiting factor for the restriction of Prickle2 levels. (a) Western blot analysis of ubiquitination of Prickle2. GFP-Prickle2 was co-expressed with Vangl2 (4th lane) or Cullin-1 (3rd lane), and immunoprecipitated using anti-GFP antibodies. The immunoprecipitates were analysed by western blot using anti-ubiquitin antibodies. (b) Bar graphs showing the ratio of the amount of precipitated ubiquitin (parenthesis in a) to that of Prickle2. The average of the ubiquitin to Prickle ratios of the control samples was set as one arbitrary unit on the Y-axis. (c) Western blot analysis of the lysates of the cells expressing shRNA plasmid against Cullin-1 (2nd lane) or control shRNA plasmid (1st lane). (d) Bar graphs showing the relative expression levels of Prickle2 calculated from the signal intensities in (c). The average of the intensities of the control sample was set as one arbitrary

unit on the Y-axis. pcDNA3.1 was mixed as the control vector to adjust the total amount of the transfected plasmid DNA. Significant differences versus the control groups were calculated using one-way ANOVA ($p=0.029$) followed by Tukey's multiple comparisons test (b) or Student's t test (d) is marked with * (*; $p<0.05$). a.u.: arbitrary unit.

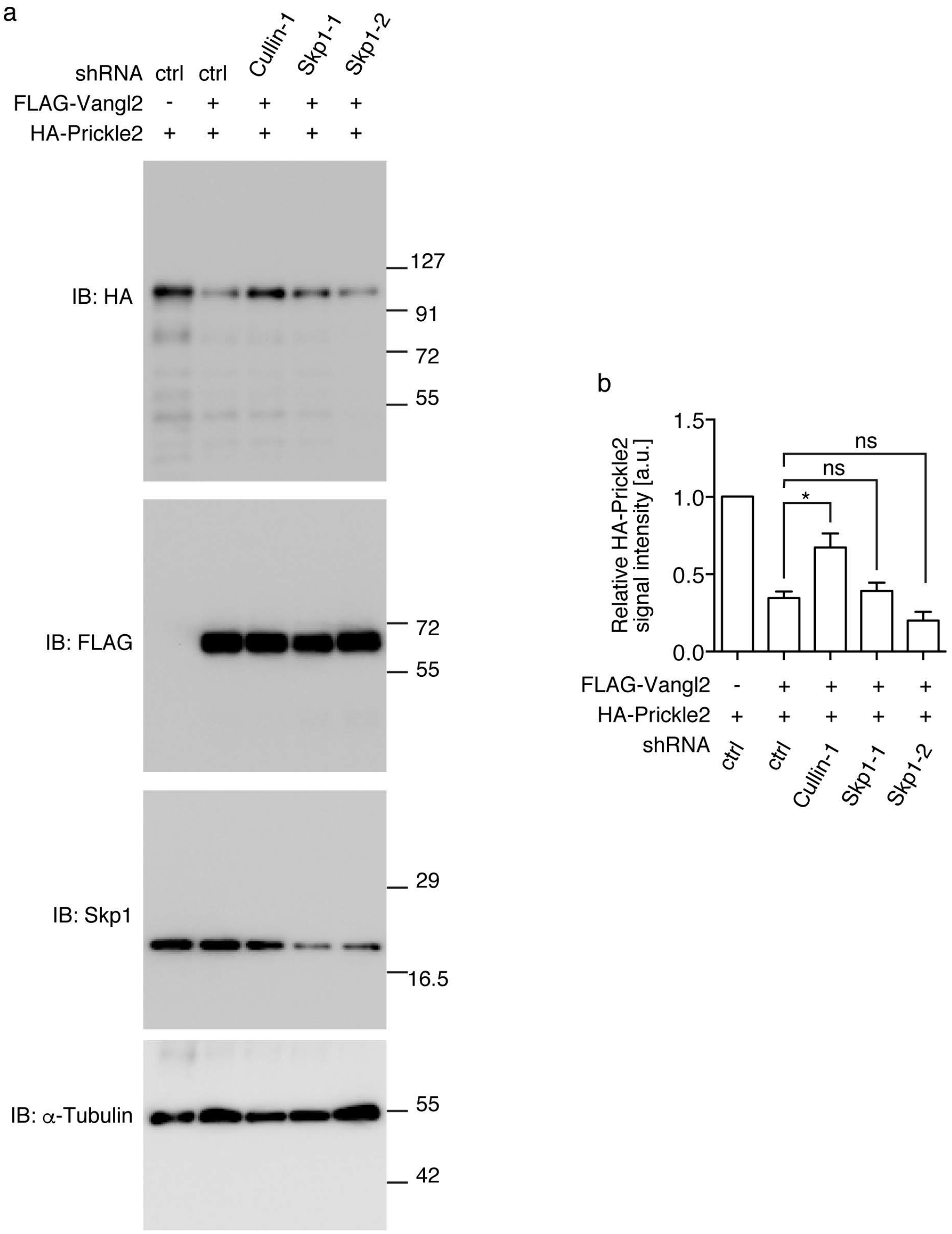

Supplementary Figure S4

Inhibition of Skp1 in the Vangl2 induced reduction in Prickle2 levels. (a) Western blot analysis of HEK293T cells expressing the indicated plasmids. Total cell lysates were immunoblotted with anti-HA (Prickle2), anti-FLAG (Vangl2), anti-Skp1, or anti-α-tubulin. (b) Bar graphs showing the quantitation of the signal intensities of the western blots shown in (a). The average Prickle2 levels expressed in cells transfected with HA-tagged wild type Prickle2 were set as one arbitrary unit on the Y-axis. Note the insignificant recovery of Prickle2 levels in cells transfected with shRNA-Skp1-1 or-2. pCS2FLAG was mixed as the control vector to adjust the total amount of the transfected plasmid DNA (a). Significant differences versus the control group calculated using one-way ANOVA ($p<0.0001$) followed by Tukey's multiple comparisons test is marked with * (*; $p<0.05$). a.u.: arbitrary unit.

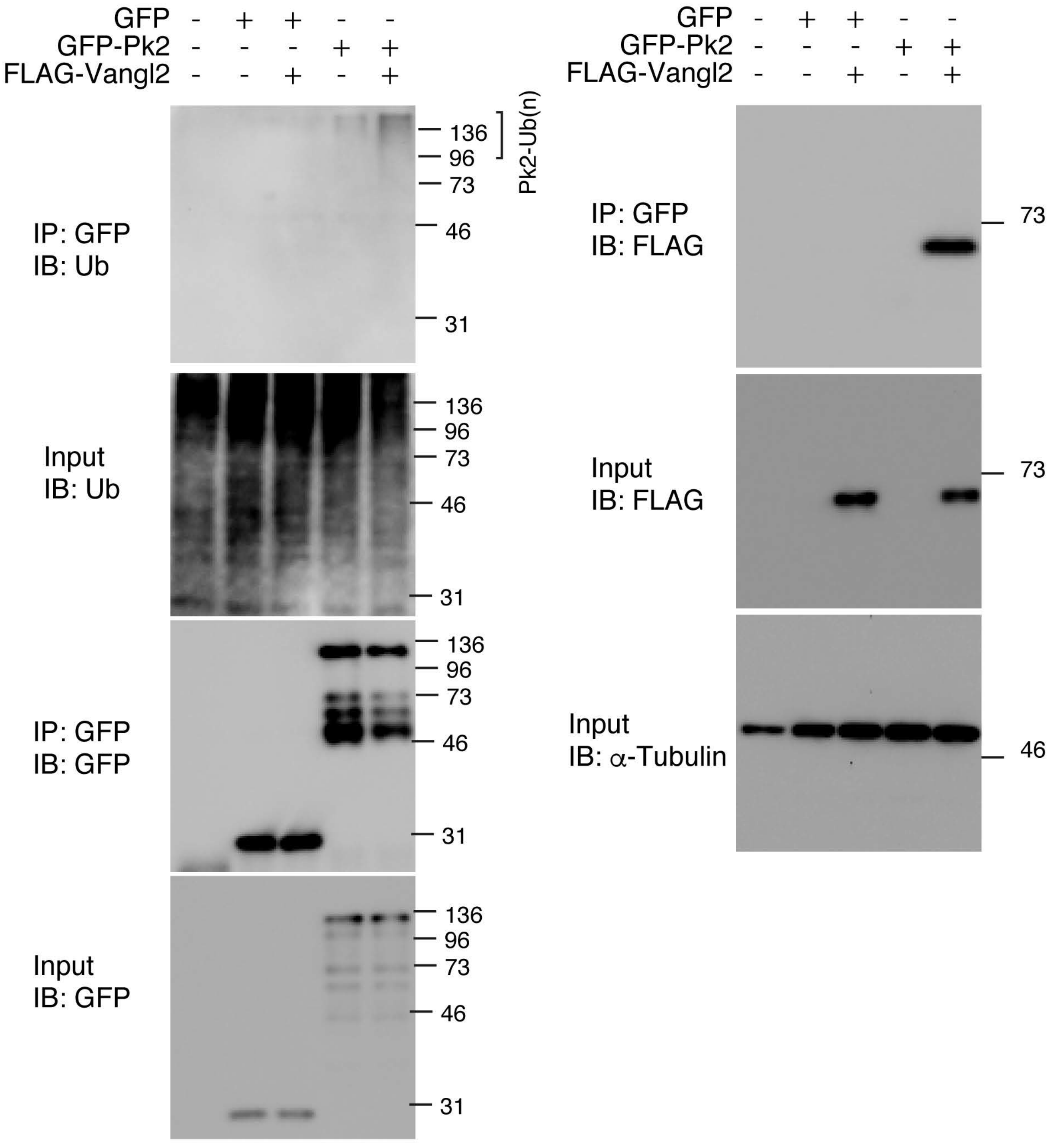

Supplementary Figure S5

Ubiquitination of GFP is not enhanced by co-expression of Vangl2. The GFP-Prickle2 (4th and 5th lanes) or GFP-tag expression plasmid alone (2nd and 3rd lanes) was transfected to the HEK293T cells with (3rd and 5th lanes) or without (2nd and 4th lanes) FLAG-Vangl2. Total cell lysates were analyzed by immunoprecipitation and/or western blotting. pcDNA3.1 was mixed as the control vector to adjust the total amount of the transfected plasmid DNA. Note the lack of ubiquitination of control GFP by Vangl2 in the experimental condition that GFP-Prickle2 is ubiquitinated by the Vangl2 expression.

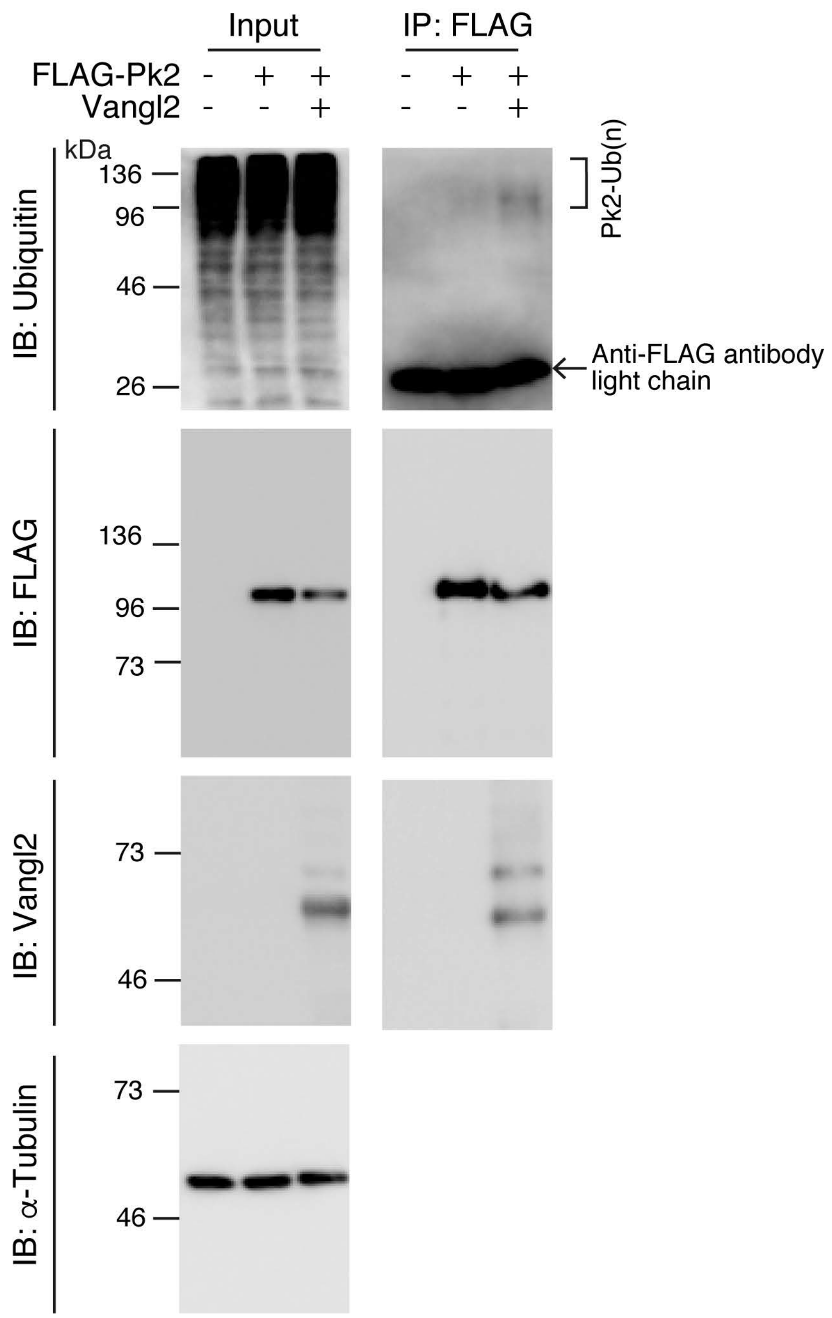

Supplementary Figure S6

Ubiquitination of FLAG-tagged Prickle2 is also enhanced by co-expression of Vangl2. The FLAG-Prickle2 expression plasmid was transfected with (3rd lane) or without (2nd lane) that of WT Vangl2. Total cell lysates were analyzed by immunoprecipitation and/or western blotting using the indicated antibodies. Note the reduced expression and increased ubiquitination of FLAG-Prickle2 by the addition of WT Vangl2. pcDNA3.1 was mixed as the control vector to adjust the total amount of the transfected plasmid DNA.

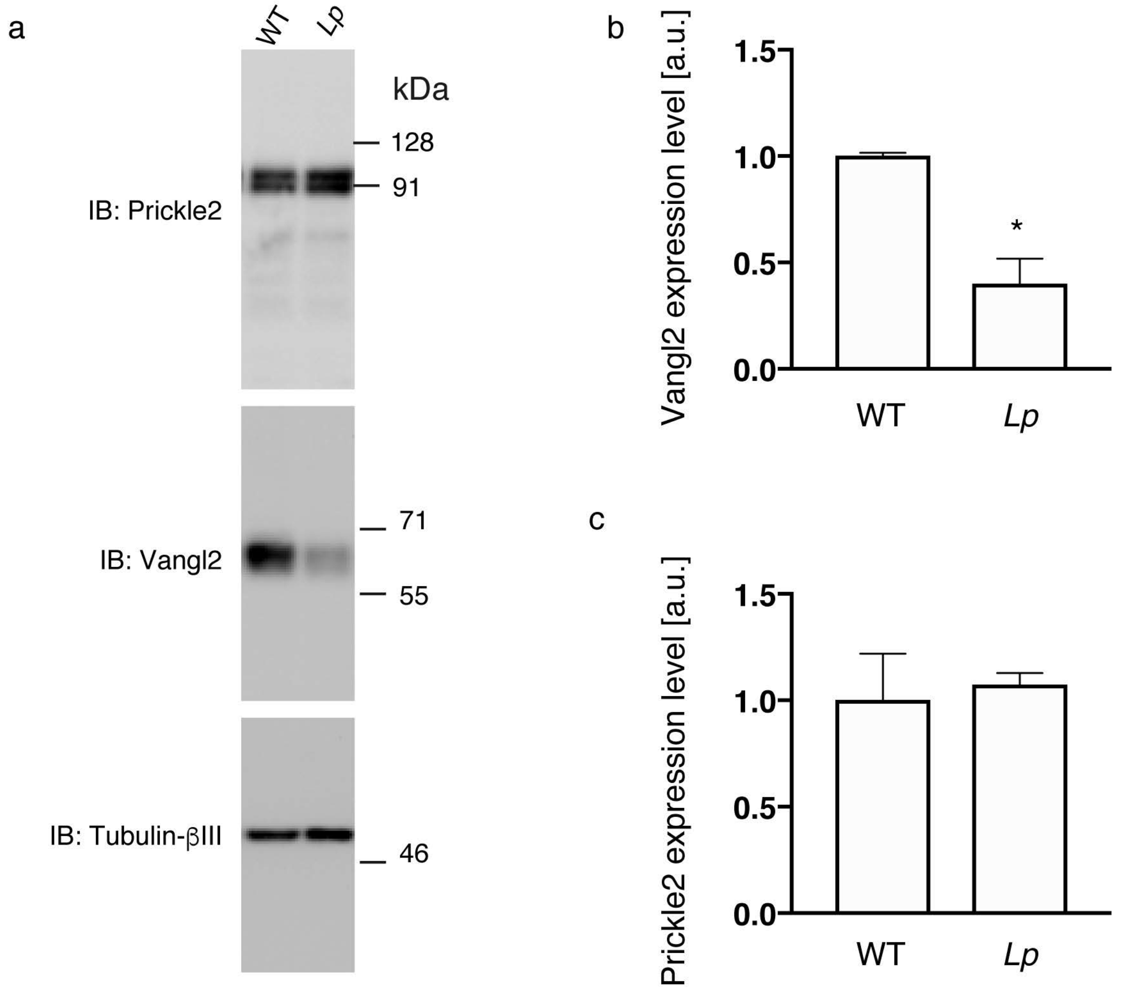

Supplementary Figure S7

Lack of increased Prickle2 protein expression levels in the brain tissues of the *Lpt* mutant mice. The brain tissues dissected out from the perinatal male mice were homogenised using the cell lysis buffer. The whole-tissue lysate containing 5 μg of protein was loaded per gel lane of SDS-PAGE and subjected to western blot analysis (a). A representative result was shown for each genotype. (b, c) Bar graphs showing the quantitation of the signal intensities of the western blots shown in (a). The average of the intensities of the control sample was set as one arbitrary unit on the Y-axis. Note that Prickle2 expression levels were not significantly increased, although those of Vangl2 were markedly reduced in the *Lpt* mutant brains. Significant differences between *looptail* (*Lp*) and wild type (WT) were calculated using Student's t test is marked with * (*; $p<0.05$). a.u.: arbitrary unit.

Figure 1b

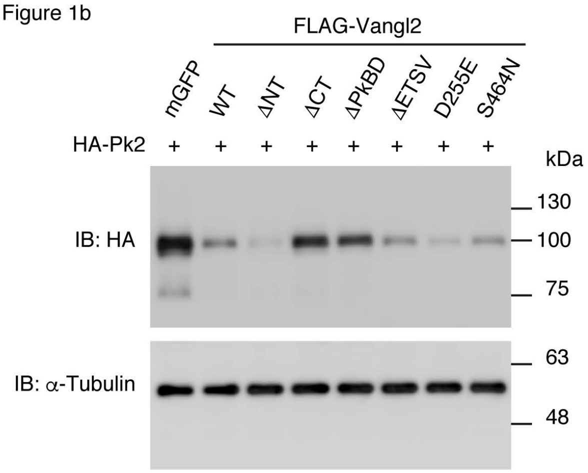

Figure 1d

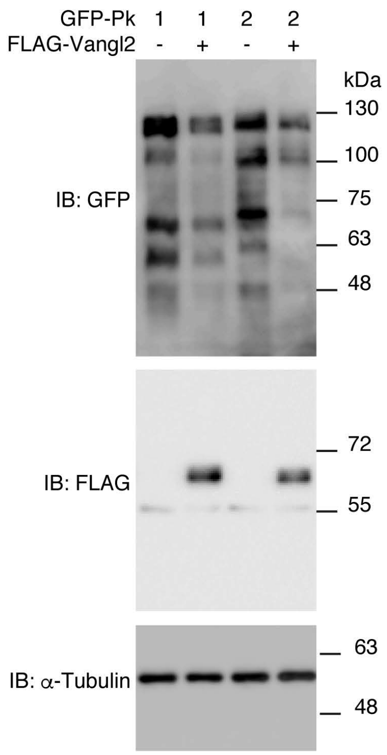

Figure 2a

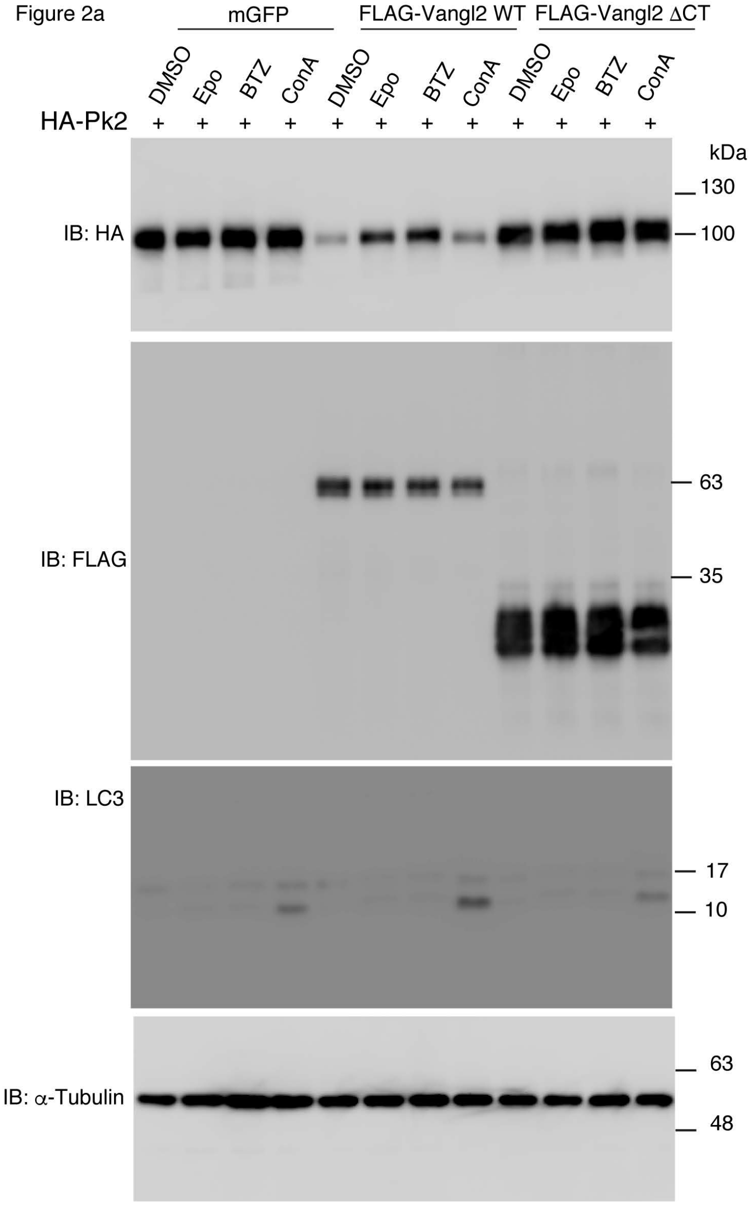

Figure 2e

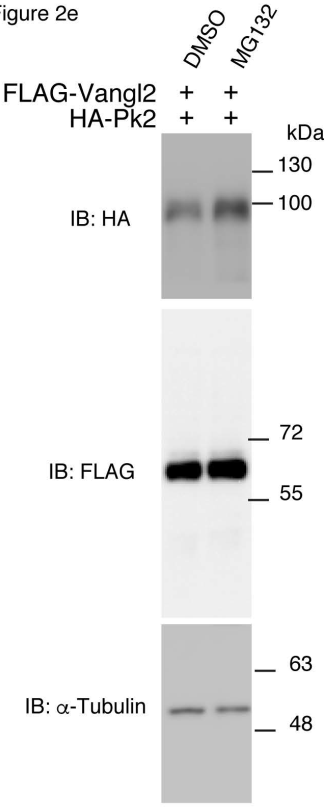

Figure 3a

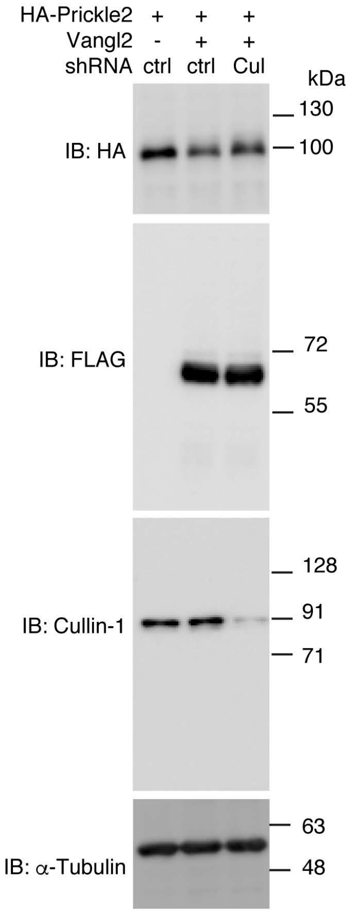

Figure 3c

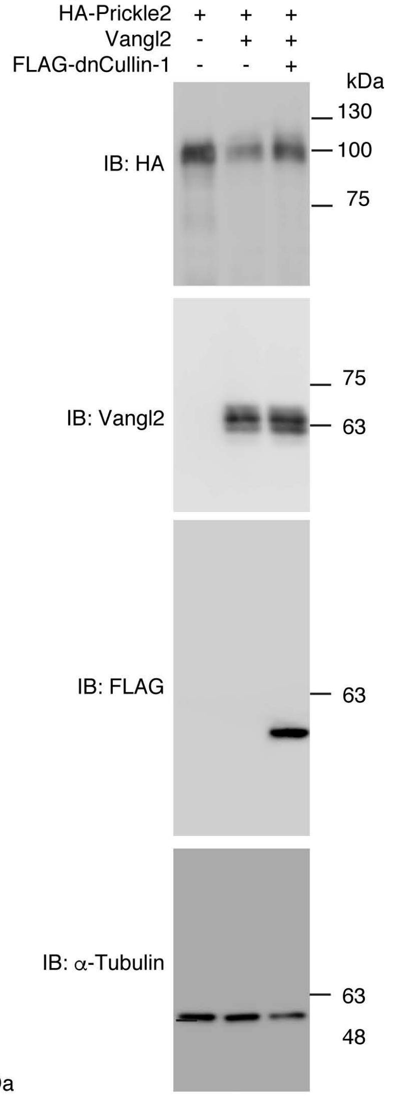

Figure 3i

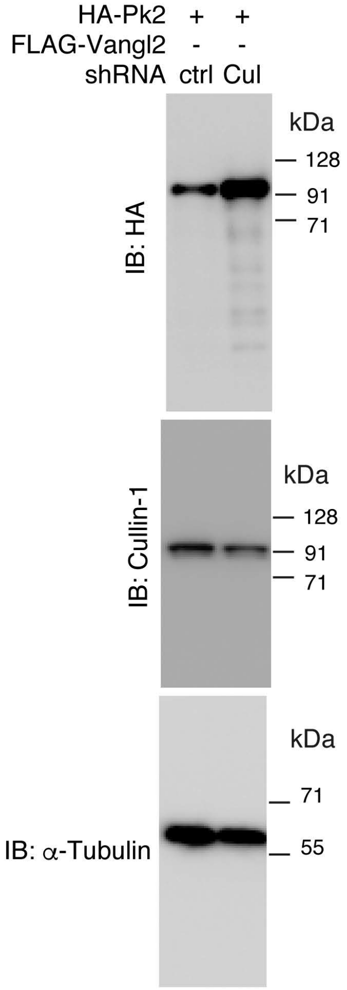

Figure 3e

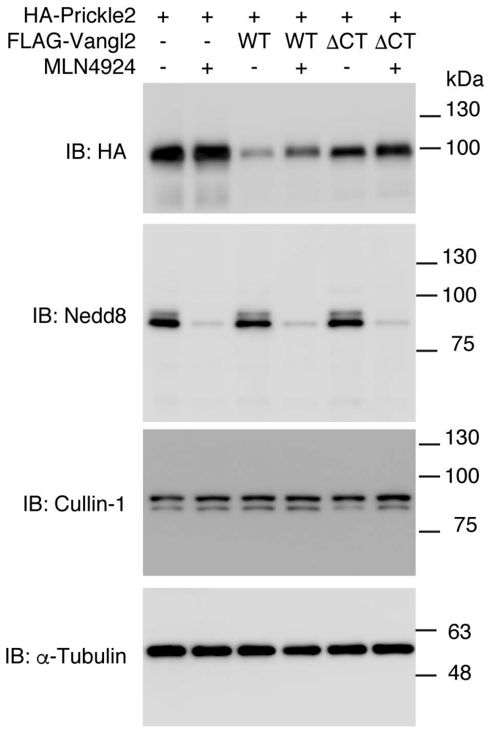

Figure 4a

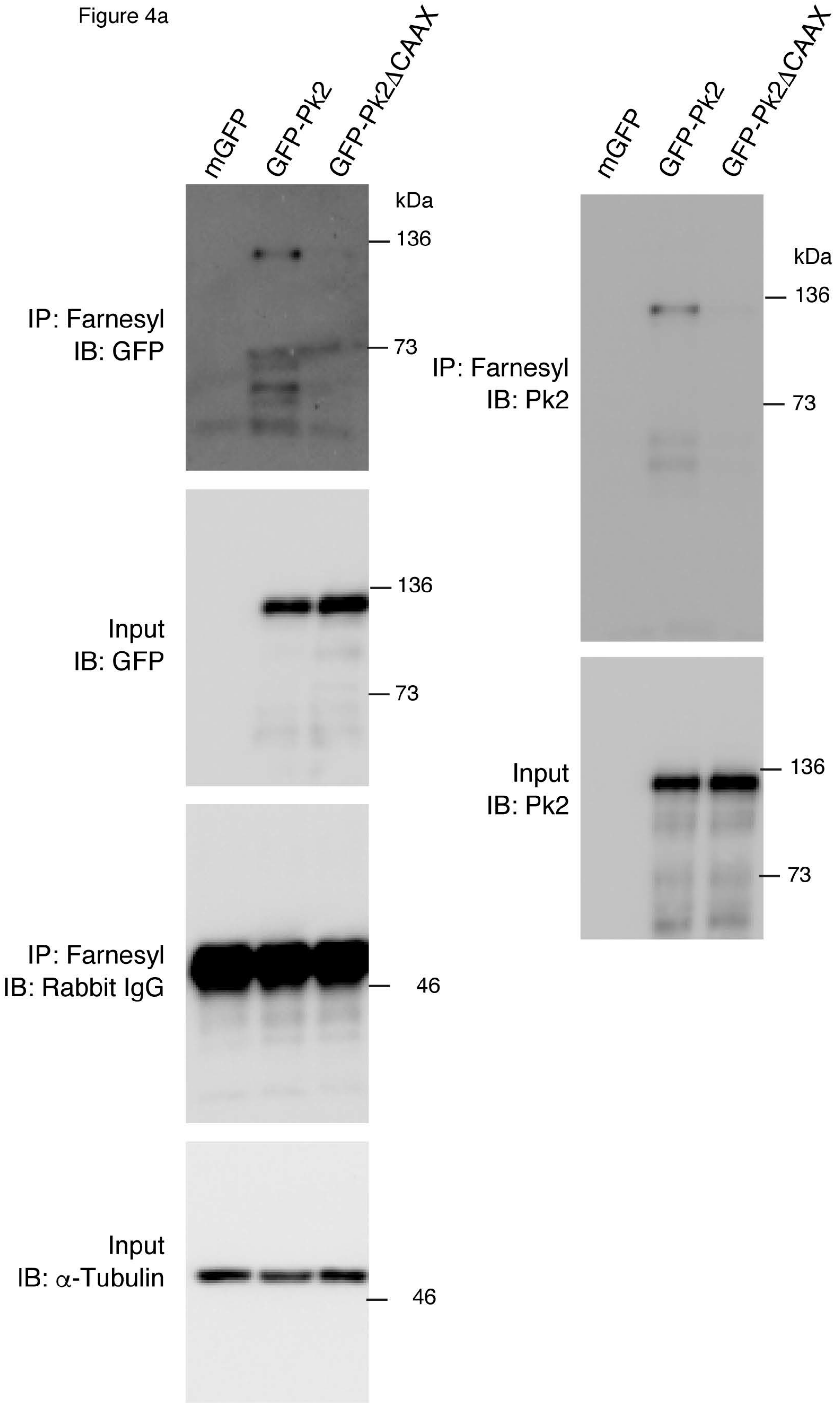

Figure 4b

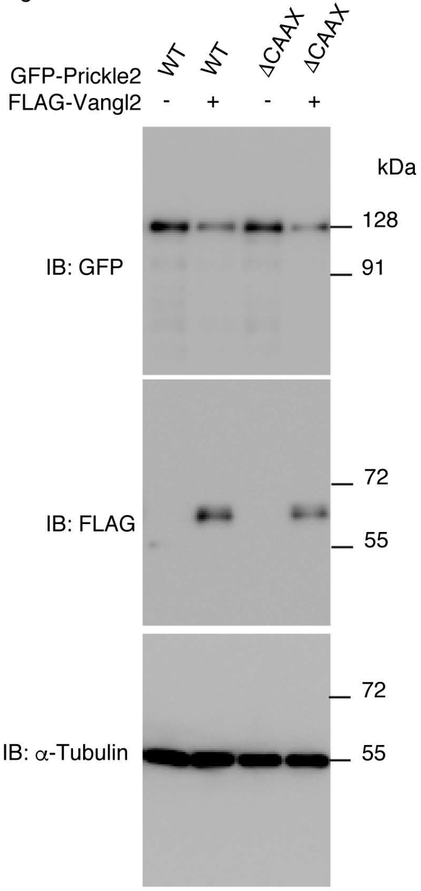

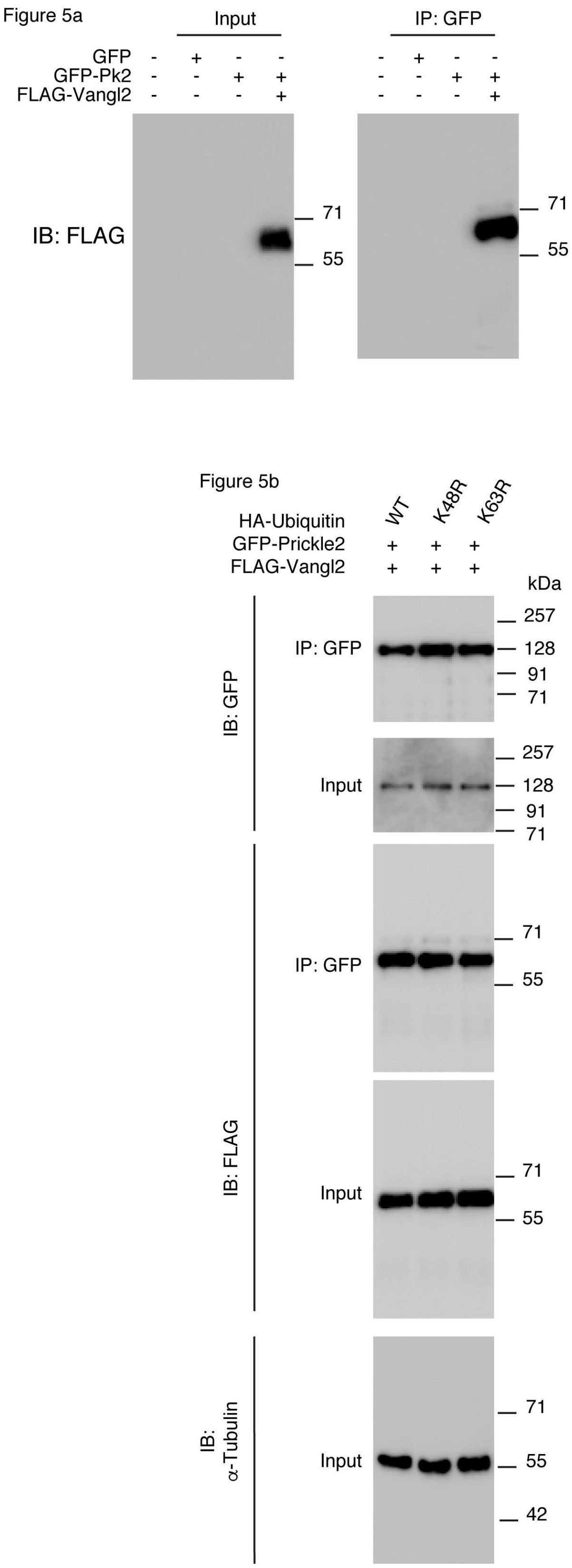

Supplementary Figure S8

Uncropped images of the western blot analyses used in Figures 1-5.

Supplementary Tables

Clone Name	GeneBank ID	Details	Vector	Used primer (5'->3')
CUL-1 shRNA		Annealed oligonucleotides ligated into AgeI-BamHI site.	FCG	CCGGGCAAAGTGCTGAATGGAATTTCAAGAGAATTCCATTCAGCACTTTGCCCTTTTTT, GATCAAAAAAGGGCAAAGTGCTGAATGGAATTCTCTTGAAATTCCATTCAGCACTTTGC
FLAG-Vangl2	BC052195	ref.16	pCS2+	
FLAG-Vangl2DETSV		ref.16	pCS2+	
FLAG-Vangl2 DNT		ref.16	pCS2+	
FLAG-Vangl2 DCT		ref.16	pCS2+	
FLAG-Vangl2 DPkBD		ref.16	pCS2+	
FLAG-Vangl2 DETSV		ref.16	pCS2+	
FLAG-Vangl2 D255E		ref.16	pCS2+	
FLAG-Vangl2 S464N		ref.16	pCS2+	
3xFLAG-Prickle2		Addgene #24645	pGateway	
GFP-Prickle2	NM_001081146	N-terminal Venus-tagged Vangl2. PCR fragment ligated into the EcoRI-SalI site.	pVenus-C1	
GFP-Prickle2DCAAX		S-farnesylation motif sequence was deleted by in-fusion HD	pVenus-C1	CAAAAACTAAGGATCCACCGGATCTAGATA, GATCCTTAGTTTTTGCTCTTCTGTCTTTT
HA-Prickle2	NM_001107876	ref.20	pCS2+	
mGFP		ref.20	pCI	
HA-Ubiquitin	NM_001281716	Addgene #18712	pcDNA3	
HA-Ubiquitin K48R		K48R mutagenesis using in-fusion HD	pcDNA3	GCAGGCAGGCAGCTGGAAGATGGCCGT, CAGCTGCCTGCCTGCAAAGATGAGCCT
HA-Ubiquitin K63R		K63R mutagenesis using in-fusion HD	pcDNA3	ATCCAGAGGGAGTCGACCCTGCAC, CGACTCCCTCTGGATGTTGTAGTC
Skp1 shRNA-1		Annealed oligonucleotides ligated into AgeI-BamHI site.	FSΔV	CCGGCTGAAGATGATGAGAACAATTCAAGAGATTGTTCTCATCATCTTCAGCCTTTTTT, GATCAAAAAAGGCTGAAGATGATGAGAACAATCTCTTGAATTGTTCTCATCATCTTCAG
Skp1 shRNA-2		Annealed oligonucleotides ligated into AgeI-BamHI site.	FSΔV	CCGGGAGTTCTGATGGAGAGATATTCAAGAGATATCTCTCCATCAGAACTCCCTTTTTT, GATCAAAAAAGGGAGTTCTGATGGAGAGATATCTCTTGAATATCTCTCCATCAGAACTC

Supplementary Table S1
Detailed information on the plasmids used in this study.

Antigen	Host	Manufacturer	Clone or Product ID	Application (Dilution)	Conjugation
a-Tubulin	Rabbit	Abcam	ab15246	IB (1:500)	
c-Myc	Mouse	Santa Cruz	9E10	IB (1:500)	
beta III Tubulin	Rabbit	Abcam	EPR1569Y	IB (1:5000)	
Cullin-1	Rabbit	Abcam	EPR3103Y	IB (1:2000)	
Farnesyl	Rabbit	Millipore	AB4073	IP (1:300)	
FLAG	Mouse	Wako	1E6	IB (1:2000)	
GFP	Rabbit	MBL	A6455	IP (1:300)	
GFP	Rat	Nacalai	GF090R	IB (1:1000)	
Goat IgG	Donkey	Santa Cruz	sc-2020	IB (1:5000)	HRP
HA	Rat	Roche	3F10	IB (1:1000)	
LC3	Mouse	MBL	8E10	IB (1:1000)	
Mouse IgG	Goat	Bio-Rad	170-6516	IB (1:5000)	HRP
Mouse IgG Light Chain specific	Goat	Jackson ImmunoResearch	115-035-174	IB (1:5000)	HRP
Nedd8	Rabbit	Abcam	Y297	IB (1:1000)	
Prickle1	Goat	Santa Cruz	sc-69225	IB (1:1000)	
Prickle2	Rabbit		T27	IB (1:2000)	
Rabbit IgG	Goat	Santa Cruz	sc-2030	IB (1:5000)	HRP
Rabbit IgG Light Chain specific	Mouse	Jackson ImmunoResearch	211-032-171	IB (1:5000)	HRP
Rat IgG	Goat	Santa Cruz	sc-2065	IB (1:5000)	HRP
Skp1	Rabbit	Abcam	EPR3304	IB (1:1000)	
Ubiquitin	Mouse	Santa Cruz	P4D1	IB (1:500)	
Vangl2	Goat	Santa Cruz	N-13	IB (1:250)	
Vinculin	Mouse	Santa Cruz	H-10	IB (1:500)	

Supplementary Table S2
Detailed information on the antibodies used in this study.
